# Prolactin enhances T regulatory cell promotion of breast cancer through the long form prolactin receptor

**DOI:** 10.1016/j.tranon.2021.101195

**Published:** 2021-08-07

**Authors:** Kuan-Hui Ethan Chen, Mrinal Ghosh, Lorena Rivera, Samuel Lin, Anil Kumar, Srividya Swaminathan, Mary Y. Lorenson, Ameae M. Walker

**Affiliations:** a Division of Biomedical Sciences, University of California, Riverside, CA 92521, United States; b Department of Systems Biology, Beckman Research Institute, City of Hope, Duarte, CA 91010, United States

**Keywords:** Prolactin receptor knockdown, Prolactin, Tumor Treg recruitment, Epithelial to mesenchymal transition, TGF-β1, IL-10, CTLA-4, PRLR, prolactin receptor, LFPRLR, long form prolactin receptor, SMO, splice modulating oligomer, GFP, green fluorescent protein, Treg, T regulatory cell, Teff, T effector cell

## Abstract

Previous work has shown systemic knockdown of the long form prolactin receptor (LFPRLR) *in vivo* markedly reduced metastasis in mouse models of breast cancer, but whether this translated to prolonged survival was unknown. Here we show that LFPRLR knockdown in the highly metastatic, immunocompetent 4T1 model prolonged survival and reduced recruitment of T regulatory cells (Tregs) to the tumor through effects on the production of CCL17. For the Tregs still recruited to the primary tumor, LFPRLR knockdown both directly and indirectly reduced their ability to promote tumor parenchymal epithelial to mesenchymal transition. Importantly, effects of prolactin on expression of mesenchymal genes by the tumor parenchyma were very different in the absence and presence of Tregs. While systemic knockdown of the LFPRLR downregulated transcripts important for immune synapse function in the remaining tumor Tregs, splenic Tregs seemed unaffected by LFPRLR knockdown, as demonstrated by their continued ability to suppress anti-CD3/CD28-stimulated effector cell proliferation at 1–5 months. These results demonstrate that knockdown of the LFPRLR achieves intra-tumor immunotherapeutic effects and suggest this occurs with reduced likelihood of peripheral inflammatory/autoimmune sequelae.

## Highlights

• Systemic knockdown of the long form prolactin receptor *in vivo* increases survival in an aggressive, immunocompetent model of stage IV, triple negative breast cancer.
• Knockdown of the long form prolactin receptor reduces Treg recruitment to tumors by reducing tumor parenchymal production of CCL-17.
• Those Tregs still recruited to primary tumors have a substantial reduction in their ability to promote epithelial to mesenchymal transition of tumor parenchyma.
• For the Tregs in the primary tumor, there is transcript downregulation of components of the T cell receptor complex and CTLA-4.
• Tregs outside of the tumor have normal ability to suppress T effector cell proliferation after 1–5 months of treatment.
• Knockdown of the long form of the prolactin receptor therefore seems to have an intra-tumor immunotherapeutic effect without effect on peripheral Treg function.

## Introduction

The extra-parenchymal tumor microenvironment includes a complex extracellular matrix, many types of stromal cells, and local accumulations of cytokines and chemokines produced by both parenchymal and stromal cells, sometimes sequestered by components of the extracellular matrix. Stromal cells that may contribute to the progression of cancer include fibroblasts, endothelial cells and various immune cells [1]. Immune cells, including antigen-presenting cells and effector lymphocytes, initially mount a response against transformed cells, but with continued inflammation there is recruitment of T regulatory cells (Tregs) that dampen the response, allowing the tumor cells to flourish [2]. Thus, accumulation of Tregs is associated with a worse prognosis in almost all cancer types [3], [4], [5], [6].

In addition to preventing immune clearance of tumor cells, Tregs also play other important roles in tumor progression, particularly in metastasis [7], [8], [9], [10]. Because of these roles, therapeutics targeting Tregs are proving to be effective treatments for many cancers. However, several studies have indicated that targeting Tregs with current immunotherapeutics can result in development of a variety of inflammatory disorders and autoimmune diseases [11], [12], [13], [14], [15], [16], an issue considered in the current study.

Although best known for its role in lactation, prolactin is also known to promote the development and progression of breast and other cancers [17], [18], [19]. Elevated serum prolactin levels and increased expression of the long form prolactin receptor (LFPRLR) on tumor parenchyma are associated with higher risks of cancer progression [20], [21], [22]. In addition, prolactin is an autocrine/paracrine factor that promotes tumor cell survival, proliferation and invasion [18,19,23]. Recently, we and others showed that prolactin promotes tumor metastasis *in vivo* [24,25]; in our study, we more specifically showed it was prolactin working through the long rather than short forms of the PRLR [24]. However, whether this was a direct effect of prolactin on tumor parenchyma or through stromal cells, or both, was not determined.

The prolactin receptor (PRLR) belongs to the type I cytokine receptor family and prolactin has effects on many, if not all, leukocytes [26], [27], [28], [29], [30], [31]. Intriguingly, and unlike other immune cells that only express the PRLR upon activation, Tregs constitutively express the PRLR [32], thereby suggesting constant modulation of Treg function by prolactin.

Here we demonstrate the effect of systemic knockdown of the LFPRLR on Treg-parenchymal interactions in an aggressive, syngeneic breast cancer model. We report increased survival of these immunocompetent animals and decreased representation of Tregs in the tumor stroma. For the residual tumor Tregs, we report almost complete downregulation of expression of molecules involved in the production of an immune synapse, and a decreased ability to promote epithelial to mesenchymal transition (EMT), all accomplished without effect on the ability of peripheral Tregs to suppress effector cell proliferation.

## Materials and methods

### Splice-modulating oligomers (SMO)

The splice-modulating and control oligomers, linked to octaguanidine dendrimers for cell/tissue penetration ability *in vivo,* were custom synthesized by Gene Tools (Philomath, Oregon). The sequences of the LFPRLR SMOs were designed to bind to the intron-exon junctions specific to the LFPRLR in mouse or human, as described and characterized previously [24]. The control oligomer [24] has no binding to DNA or mRNA in human or mouse.

### Animals

Female, immunocompetent Balb/c mice with EGFP expression under the control of the Foxp3 promoter (#0,006,769), immunodeficient NOD-SCID (NOD.Cg-*Prkdc^scid^*/J) mice (#001,303), and hyperprolactinemic, lupus-prone MRL-lpr (MRL/MpJ-*Fas^lpr^*/J mice (#000,485) were obtained from Jackson Labs, Sacramento, CA. The study was limited to female animals because of the 100:1 ratio of breast cancer in females *versus* males. All animal experimentation was approved by the University of California Institutional Animal Care and Use Committee.

### Tumor cell lines

The mouse (4T1, ATCC CRL-2539^TM^) and human (BT-474, ATCC HTB-20) breast cancer cell lines, newly purchased from ATCC to ensure their authenticity, were routinely cultured in RPMI 1640 supplemented with 10% FBS. To ensure expression of the PRLR, cells used for all studies were under 15 passages and in most experiments were below passage 7.

### Survival trials

Twenty-five hundred 4T1 syngeneic or human BT-474 breast cancer cells were suspended in 50% Matrigel (BD biosciences, #354,248) in a volume of 50 µl and gently injected into the fourth mammary fatpad of 8-week old Foxp3^+^EGFP Balb/c or NOD-SCID mice, respectively, as described previously [24]. Treatment utilized 28-day Alzet® minipumps (#2004, Durect, Cupertino, CA), began on day 2 after cell inoculation, and pumps were replaced as needed. The pumps delivered 100 pmoles/h/mouse of either the Control SMO or LFPRLR SMO (mouse or human sequence, as appropriate). As described previously [24], mice receiving BT-474 cells (both Control SMO- and PRLR SMO-treated) also received recombinant human PRL. In some trials, primary tumors were removed when they became palpable. Animal health was recorded daily, and the experiment was terminated when there was only 30% survival in the control group. Animals in control and PRLR SMO groups were randomly selected, coded by ear marks, housed together 5/cage, and handled daily.

### Analysis of tumor Tregs

To ensure an adequate number of tumor Tregs for analysis, 10 million 4T1 cells were injected into 8-week old Foxp3^+^EGFP Balb/c mice, as above, and tumors were harvested at 12 or 28 days after inoculation. Treatment with Control SMO or LFPRLR SMO was at the same dose of 100 pmoles/h/mouse. Tumors were dissociated with Accutase (Innovative Cell Technologies Inc, # AT-104) prior to flow sorting or cytometry.

### Flow cytometric analyses

Based on forward and side scatter characteristics, live cells were gated. For analysis of surface and intracellular markers, fluorescence minus one (FMO) and isotype controls were used for gate setting, respectively. Fc blocking and directly conjugated antibodies were used to distinguish CD4^+^and CD8^+^cells and Tregs. Tregs were identified as CD4^+^, CD25^+^ and EGFP^+^, with the EGFP fluorescence derived from activity of the Foxp3 promoter. Pilot studies indicated that LFPRLR SMO treatment downregulated CD3 in tumor Tregs. Gating therefore began with CD4 positivity in order not to underestimate Tregs. Cells are therefore reported as CD4^+^ or CD8^+^ cells and not as CD4^+^or CD8^+^*T* cells.

For analysis of cytokine production, cells were pretreated with 0.0026% Golgistop (BD Biosciences #554,724) for 4 h to block cytokine release before surface marker staining. Cells were then fixed, permeabilized (BD Bioscience 554,722) and stained with fluorophore-conjugated antibodies specific for each cytokine.

All antibody information can be found in Supplementary Table I. Flow cytometry was performed on a BD FACS Canto II and data were analyzed by Flowjo software.

### RNAseq of tumors and tumor Tregs

Six Foxp3^+^EGFP Balb/c mice, inoculated with 10 million cells, were treated with Control SMO and 6 with LFPRLR SMO for 12 days. Total RNA was extracted from primary tumors with Trizol (Invitrogen # 15,596,018) and quantified/assessed for purity by nanodrop. Equal amounts of RNA from each animal were combined to a single tube per treatment. The rRNA was then depleted (New England Biosciences E6310S) and the cDNA library constructed (Kapa Biosystems KK8400). The constructed Control SMO and LFPRLR SMO cDNA libraries were multiplexed and subjected to Illumina MiSeq analysis. Using the statistical environment R (R Core Team, 2014), expression values were extracted from .gtf files using the hisat2/stringtie [33].

For RNAseq of tumor Tregs, Tregs were sorted on the basis of Foxp3 positivity from an additional set of accutase-dissociated primary tumors from 12-day Control SMO- or LFPRLR SMO-treated Foxp3^+^EGFP Balb/c mice (*n* = 6 for each treatment) and RNA extracted and processed, as above. The RNAseq results of both whole tumor (including Tregs) and tumor Tregs alone are available on NCBI Gene Expression Omnibus (GEO) with the accession number GSE111329.

### Treg migration assay

4T1 cells were incubated in serum-free RPMI 1640 medium (5 × 10^6^ in 7 ml) with or without 100 ng/mL prolactin for 1 h and the conditioned medium collected as the chemoattractant for a Transwell™ assay. Ten million splenocytes, isolated from Foxp3^+^EGFP Balb/c mice were placed in the upper chamber (5 μm pore size, Corning 3421) and 1 mL conditioned medium from 4T1 cells with or without prolactin treatment was added to the lower chamber in the presence or absence of 1 μg/mL mouse anti-CCL17 or anti-CCL22 antibody (R & D systems, AF529, AF479, respectively). Cells that migrated into the lower chamber in 1 h were collected and analyzed by flow cytometry. Treg numbers were deduced from the percentage of CD4^+^ cells that were EGFP^+^.

### Co-culture of Tregs to examine epithelial to mesenchymal transition

Tumor Tregs from an additional set of animals, sorted as EGFP^+^ cells from Foxp3^+^EGFP BALB/c mice, were *ex vivo* treated with 1 μM Control SMO or LFPRLR SMO for 48 h in RPMI supplemented with 10% FBS. The medium containing the SMO was then removed by pelleting and resuspension of the Tregs. Ten thousand Tregs were transferred to each well of a 96-well plate and co-cultured with 10,000 4T1 cells in the presence or absence of 100 ng/mL recombinant prolactin in serum-supplemented medium for 48 h. Tregs were then washed away and the adherent 4T1 cells were processed to examine expression of mesenchymal markers by quantitative real-time PCR (qRT-PCR).

In other experiments, tumor Tregs were sorted from *in vivo* Control SMO- or LFPRLR SMO-treated Foxp3^+^EGFP Balb/c mice. Ten thousand Tregs were then co-cultured directly with an equal number of 4T1 cancer cells for 48 h. Tregs were then washed away and the adherent 4T1 cells were processed to examine expression of the same EMT markers. qRT-PCR was performed using a Biorad CFX system and results were normalized to *Gapdh*. Primer sequences are listed in Supplementary Table II.

### Treg suppression assay

Foxp3^+^ Tregs and Foxp3^−^ splenocytes were sorted from spleens of non-tumor bearing Foxp3^+^EGFP Balb/c mice treated for 1, 2, 3 or 5 months with Control SMO or LFPRLR SMO. Twenty thousand Foxp3^−^splenocytes were plated 1) either alone with stimulation by anti-CD3e/CD28 antibodies (5 μg/mL of anti-CD3e, BD #550,275 and 2 μg/mL of anti-CD28, BD #553,295) in the presence of IL-2 (20 IU, Sigma Aldrich #I2644), 2) with stimulation by anti-CD3/CD28 antibodies in the presence of IL-2 and 5000 Foxp3^+^ Tregs from Control SMO-treated mice, or 3) with stimulation by anti-CD3/CD28 antibodies in the presence of IL-2 and 5000 Foxp3^+^ Tregs from LFPRLR SMO-treated mice. At day 4, another 20 IU of IL-2 was added to the medium to support cell proliferation. The cells were cultured for a total of 7 days. Cells from each group were collected at day 7 and labelled with red fluorescence (PKH26, Sigma-Aldrich, MINI26). The number of red fluorescent cells reflected the final number in the different groups. Non anti-CD3/CD28-stimulated splenocytes were used as the negative control (background reading). Green fluorescence was used to measure any potential contribution to the final cell number by proliferation of Foxp3^+^ Tregs rather than effector cells. There was no difference in green fluorescence intensity after the 7-day incubation in any treatment. Thus, the difference in red fluorescence reflected the relative proliferation of splenocytes elicited by anti-CD3/CD28 stimulation in the absence or presence of Control SMO- or LFPRLR SMO-treated Tregs.

### Treatment of MRL-lpr mice

Six-week old, non-tumor bearing MRL-lpr mice were implanted with Alzet minipumps delivering the same dose of either Control SMO or LFPRLR SMO. Pumps were changed after 28 days and spleens were harvested after 56 days of treatment when the animals were 14 weeks of age.

### Statistical analyses

Kaplan Meier plots illustrated differences in survival, effects on survival were determined by the Mantel-Cox test, and the Hazard ratio was calculated by the Mantel-Haenszel method. Data illustrated by histograms are expressed as the mean ± SD. For mouse replicates, individual values are also shown. Results of pilot experiments/previous studies were used to perform power analyses. Sample size for probability of Type I error <0.05 and *Power (1-β)* >0.8 was calculated. For two sample comparisons, a simple *t-*test established significance. Multiple comparisons used ANOVA. All statistical analyses used Graphpad Prism software. Results were considered significant when *p* < 0.05.

## Results

### LFPRLR SMO treatment of immunocompetent tumor model improves survival

**Fig. 1:**
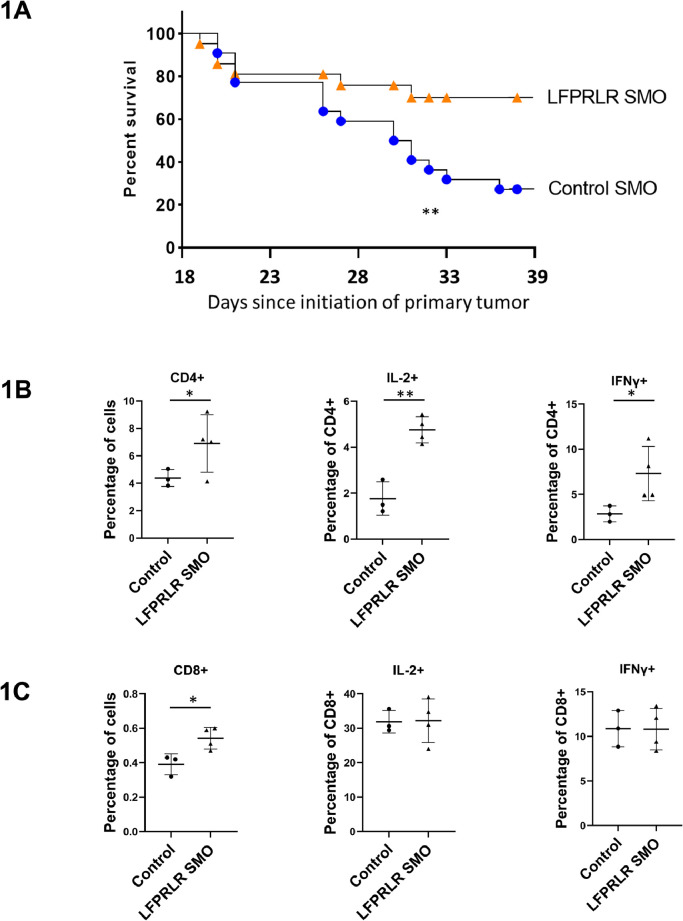
LFPRLR Knockdown Extended Survival and Enhanced Evidence of Adaptive Immune Responses to the Tumor. LFPRLR SMO treatment improved survival in the syngeneic, 4T1 Balb/c mouse model (A). Mice were orthotopically inoculated with 2500 breast cancer cells and tumors were surgically removed when palpable 14 days later. The graph shows results once deaths began to occur, which was 19 days after inoculation. After orthotopic inoculation of 10 million 4T1 breast cancer cells into Balb/c mice, treatment with LFPRLR SMO increased CD4^+^ and CD8^+^ percentages (percentages of total viable tumor cells) and the state of activation of the CD4^+^ cells within the tumor at day 28 (B&C). The results in B&C were derived from at least 3 mice per group. For the survival experiments, *n* = 21 for LFPRLR SMO and *n* = 22 for control SMO treatment. Data are presented as mean ± SD. (*p < 0.05, ** *p*<0.01).

We have previously shown that LFPRLR SMO treatment inhibited metastasis in both the 4T1 syngeneic Balb/c and BT-474 human xenograft NOD-SCID mouse orthotopic breast cancer models [24]. To determine whether this translated to an effect on animal survival, survival was examined in both models. The LFPRLR SMO improved survival in all trials in the syngeneic model but was most effective if the primary tumors were surgically removed when they became palpable (day 14 after inoculation) in a manner similar to accepted clinical treatment (Fig. 1A). The plot shows survival once deaths began to occur, which was 19 days after inoculation. However, differences in survival did not begin to appear until about day 25. The mice receiving the LFPRLR SMO had substantially improved survival (*p* value =0.02) and the Hazard ratio of LFPRLR/Control SMO was 0.36, Fig. 1A). By contrast, there was no difference in survival in the human xenograft model whether primary tumors were removed or not, or after alternative tumor initiation by transplant of an already palpable tumor (data not shown).

Although clearly there are many differences between the two models, both in the breast cancer cells themselves as well as the host mouse, one major difference is the presence *versus* absence of adaptive immunity.

### LFPRLR SMO increased effector cells and decreased Tregs in the tumors

Although we had previously shown an increased tumor infiltration by CD4^+^ and CD8^+^cells in response to LFPRLR knockdown, those studies examined tumors at a later stage (42 days) when there were many fewer cells and large-scale necrosis (24). To gain a fuller picture of what was happening at a time when the two survival curves began to diverge, we examined tumors 28 days after tumor cell inoculation. We found higher percentages of CD4^+^ cells within the tumor (percentage of combined tumor parenchymal and stromal cells) in response to LFPRLR SMO treatment. We also observed that these CD4^+^ cells were activated at a higher frequency, as assessed by their production of IL-2 and IFNγ (Fig. 1B). Similarly, CD8^+^cells constituted a higher percentage of tumor cells but, unlike more established tumors [24], the extent of their activation at this 28-day timepoint was not different between treatments (Fig. 1C). No difference in IL-2 or IFNγ mean fluorescence intensity (MFI) for the activated tumor CD4^+^ or CD8^+^ cells was observed (data not shown). While CD4^+^ and CD8^+^ cells constituted a greater percentage of the tumor, there was no general systemic effect on CD4^+^ or CD8^+^ percentages in the thymus, spleen or blood (Fig. S1A-F).

**Fig. 2:**
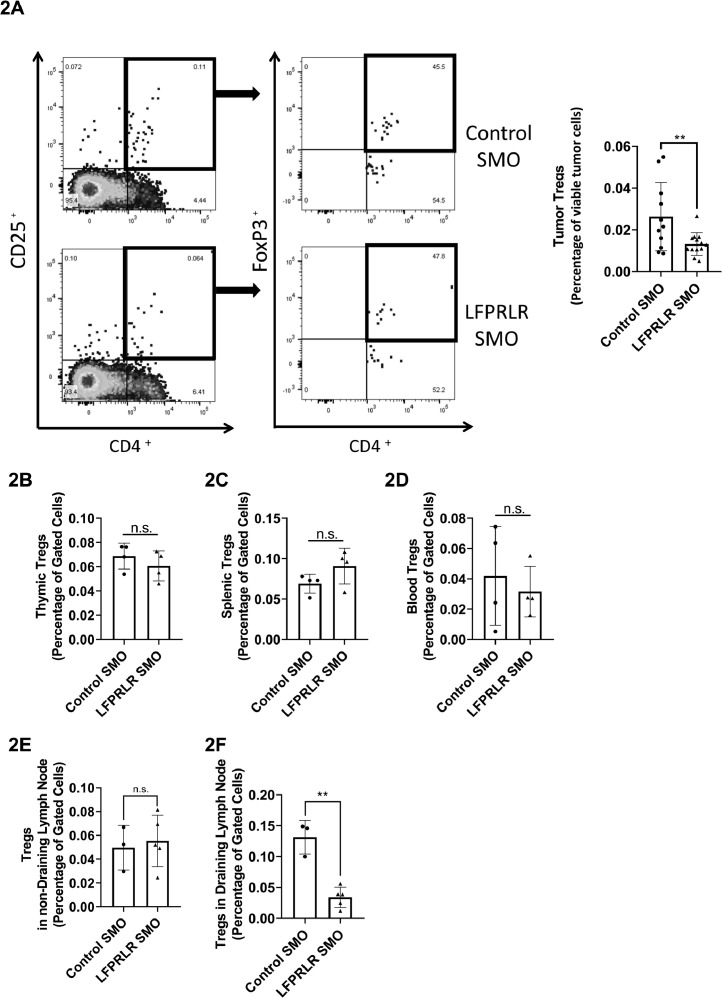
LFPRLR SMO Reduced Infiltration of Tumor by Tregs *in vivo.* Ten million 4T1 breast cancer cells were orthotopically inoculated and tumors were analyzed on day 12. The Treg percentage of viable cells in the tumor was significantly reduced in mice treated with LFPRLR SMO (A) (*n* = 13 for LFPRLR SMO and *n* = 11 for Control SMO). In a subset of these animals, Treg percentages as a function of total viable cells in the organ were examined. There were no effects of LFPRLR SMO treatment on thymic (B), splenic (C), or circulating (D) Tregs or Tregs in the non-tumor draining, contralateral inguinal lymph node (E). A consistent reduction of Tregs was found in the tumor-draining, ipsilateral inguinal lymph node (F). Data are presented as mean ± SD. (** *p*<0.01).

In contrast to total CD4^+^cells, treatment with LFPRLR SMO reduced the percentage of Tregs within the tumor microenvironment (Fig. 2A). To determine whether this was possibly secondary to systemic diminution of Tregs, we again examined the thymus, spleen, and blood. There was no evidence of changes in the percentage of Tregs in these organs in response to LFPRLR SMO (Fig. 2B-D).

With orthotopic tumor development, metastatic spread is most frequently *via* the mammary-draining lymphnode. Compared to the contralateral node, the tumor-draining lymphnode had a much higher percentage of CD4^+^ cells (82 *versus* 8%); this was not significantly impacted by LFPRLR SMO, but there was a trend towards a further increase (Fig.S1G, H).The tumor draining lymphnode also had threefold the percentage of Tregs and treatment with LFPRLR SMO normalized to contralateral lymphnode levels (Fig. 2E, F).

Treatment with LFPRLR SMO also showed a trend towards reduction of Tregs in the liver, which is a major site of metastatic spread (*p* = 0.0511). This occurred without effect on total liver CD4^+^ and CD8^+^ cells (Fig. S2A-C).

### Prolactin signaling through the LFPRLR is important to tumor Treg recruitment

**Fig. 3:**
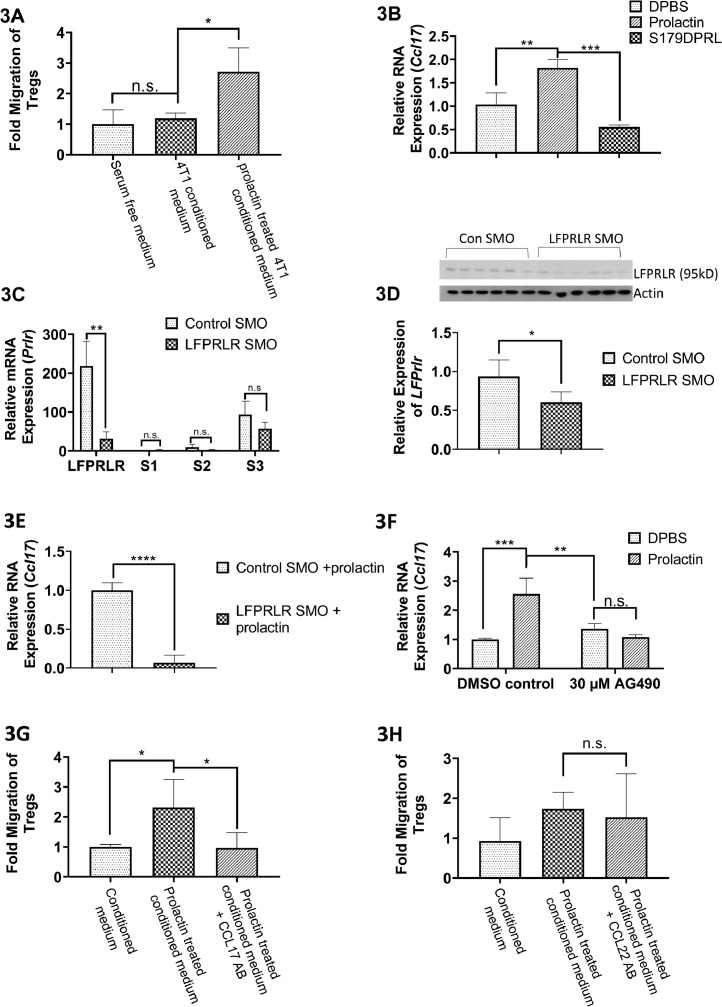
Prolactin Stimulates Treg Migration and CCL17 Is Important for Prolactin-Mediated Treg Recruitment. 4T1 cells were incubated *in vitro* in 100 ng/mL prolactin in serum-free medium for 1 h and the conditioned medium was used for the lower chamber in Transwell™ migration assays. Splenocytes from untreated Foxp3+Balb/c mice were placed in the upper Transwell™ chambers and the number of Tregs that migrated into the conditioned medium in 1 h was recorded. Conditioned medium, collected from *in vitro* prolactin (100 ng/mL)- stimulated 4T1 cells, was able to enhance migration of Tregs (A). *Ccl17* transcripts were increased in 4T1 cells after 48 h of prolactin treatment (100 ng/mL) (B). Treatment with the selective prolactin receptor modulator, S179DPRL (100 ng/mL), by contrast, reduced expression (B). An equal volume of Dulbecco's PBS was used as the vehicle control. Treatment of 4T1 cells with 1 μM LFPRLR SMO for the same period (48 h) almost completely knocked down LF*prlr* without effect on mRNA for the short forms of the receptor (C). This translated to an approximately 40% reduction in LFPRLR protein, as assessed by Western blot (D). The same pretreatment with LFPRLR SMO prevented *Ccl17* mRNA induction in the presence of prolactin (100 ng/mL, 48 h) compared with 1 μM Control SMO (E). 30 μM AG490, given as a 2 h pre-incubation, blocked prolactin-induced *Ccl17* mRNA expression (F). Addition of CCL17 antibody into the conditioned medium derived from 1 h prolactin-stimulated 4T1 cells eliminated migration of Tregs in response to conditioned medium (G), whereas an antibody against CCL22 was without effect (H). All experiments were performed 3 times using triplicate wells on each occasion (*n* = 3). For Transwell™ experiments, four independent analyses (*n* = 4) were performed. Data are presented as mean ± SD. (*< *p* 0.05, ** <*p* 0.01).

Serum-free medium, conditioned by 4T1 cells for 1 h, showed no significant chemoattractive properties for Tregs. However, with prolactin-stimulation, the 4T1 cell conditioned medium produced increased Treg migration (Fig. 3A). To determine whether prolactin, rather than substances produced by 4T1 cells in response to prolactin, was chemoattractive, Transwell™ assays were conducted with prolactin-supplemented, serum-free, non-conditioned medium. As previously-published [34], we found no chemoattractive properties of prolactin itself under these experimental conditions (not shown). Thus, prolactin stimulation of 4T1 cells results in the production of a chemoattractant for Tregs.

The known chemoattractants for Tregs are CCL17 and CCL22 [5]. As shown in Fig. 3B, prolactin stimulation of 4T1 cells *in vitro* (48 h, 100 ng/ml) induced *Ccl17* expression while the selective PRLR modulator, S179DPRL (18), had the opposite effect. There was no significant effect of prolactin on *Ccl22* expression (data not shown here but present in RNAseq results GEO: GSE111329).

When 4T1 cells were treated with LFPRLR SMO for 48 h prior to treatment with prolactin under the same conditions, LFPRLR SMO almost completely knocked down expression of the LFPRLR, with no significant effect on expression of the short forms of the receptor (Fig. 3C). This translated to an ∼40% decrease in LFPRLR protein under the same conditions (Fig. 3D). LFPRLR SMO also markedly decreased the ability of prolactin to induce *Ccl17* expression in 4T1 cells (Fig. 3E). The major signaling difference between prolactin and S179DPRL and between the LFPRLR and short forms of the PRLR that remain after LF knockdown, is use of the Jak2/Stat5 pathway by prolactin and the LFPRLR [17,18,35]. To further establish a role for PRLR signaling in *Ccl17* induction, 4T1 cells were treated with the Jak2 inhibitor, AG490, and then stimulated with prolactin. When the Jak2 inhibitor, AG490, was present in the culture medium, induction of *Ccl17* by prolactin in 4T1 cells was eliminated (Fig. 3F).

To further substantiate the role of CCL17 in prolactin-stimulated 4T1 recruitment of Tregs, an antibody against CCL17 was added to the prolactin-stimulated cancer cell-derived conditioned medium and the migration of Tregs was measured. As seen in Fig. 3G, addition of anti-CCL17 antibody eliminated the ability of prolactin treatment to increase Treg migration. When the same experiment was performed with an antibody against CCL22, there was no effect (Fig. 3H).

To link the in *vitro* work to *in vivo* conditions, whole tumors from Control SMO or LFPRLR SMO treatments were analyzed by RNAseq. A substantial, but less pronounced reduction in *Ccl17* was observed with LFPRLR SMO when analyzing the mixed cell population of the tumor rather than pure 4T1 cells (relative mean expression from 6 animals/group from 1.92 to 0.985). Once again, no reduction in *Ccl22* expression by LFPRLR SMO was observed (GEO: GSE111329). Thus, CCL17 is a major factor involved in Treg recruitment by 4T1 cells in response to physiological prolactin working through the LFPRLR.

### Tumor Tregs from LFPRLR SMO-Treated mice lost the ability to drive epithelial to mesenchymal transition of tumor cells

**Fig. 4:**
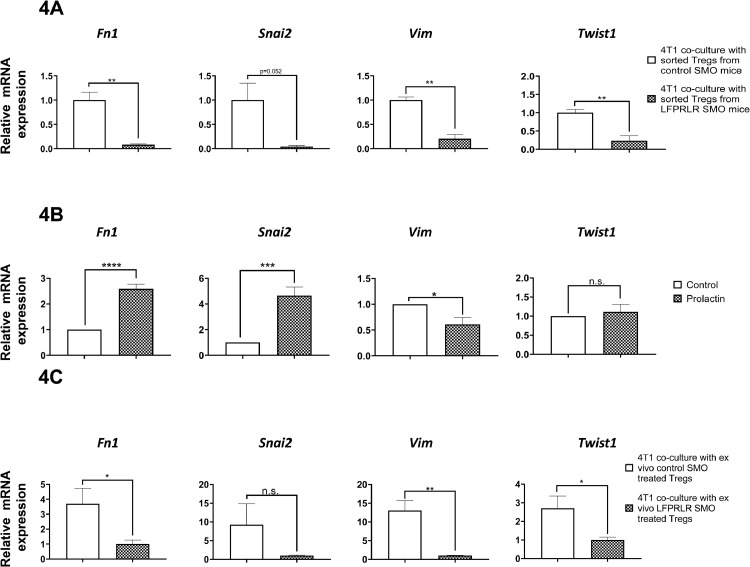
Tumor Tregs from LFPRLR SMO-Treated Mice Lost the Ability to Drive Epithelial to Mesenchymal Transition of Tumor Cells.Tumors were produced in 8-week-old female Foxp3^+^EGFP Balb/c mice by injecting 10 million 4T1 syngeneic breast cancer cells into the mammary fatpad. The mice were then treated with either the LFPRLR SMO or Control SMO for 12 days. Tumor Tregs were then sorted and ten thousand 4T1 cells were co-cultured with the same number of tumor Tregs from Control SMO- or LFPRLR SMO-treated mice. Prolactin (100 ng/mL) was added to the co-culture to mimic physiological conditions and the co-culture was performed for 48 h, after which time the Tregs were washed away and the 4T1 cells analyzed for expression of mesenchymal markers. Co-culture of 4T1 cells with tumor Tregs directly sorted from *in vivo*-treated animals showed reduced mesenchymal gene expression with tumor Tregs from LFPRLR SMO-treated mice (A). To analyze direct effects of prolactin on mesenchymal gene expression in 4T1 cells, 10,000 4T1 cells were treated with 100 ng/mL prolactin for 48 h without Tregs. Expression of *Fn1* and *Snai2* was induced by prolactin. However, the expression of *Vim* was reduced, while no effect was noted for *Twist1.* These data are derived from multiple trials in which expression in the controls was normalized to 1 (B). To determine direct effects of prolactin on tumor Tregs, Tregs were sorted by flow cytometry from untreated tumor-bearing female Foxp3 GFP^+^ Balb/c mice. The sorted tumor Tregs were then treated *ex vivo* with 1 μM Control SMO or LFPRLR SMO for 48 h. Ten thousand of these *ex vivo-*treated tumor Tregs were then co-cultured with 10,000 4T1 cells in the presence of 100 ng/mL prolactin for another 48 h. The tumor Tregs were then washed away. Like in panel A, 4T1 cells co-incubated with Control SMO-treated Tregs expressed higher mesenchymal markers (C). All co-culture experiments were performed 4 times with triplicate wells (*n* = 4) and data are presented as mean ± SD. (*p < 0.05, ** *p*<0.01).

The preceding experiments were concerned with recruitment of Tregs to tumors. Next, we asked whether there was also an effect of prolactin working through the LFPRLR on the metastasis-promoting function of Tregs. Tumor Tregs were sorted from LFPRLR SMO- or Control SMO-treated mice and then placed in co-culture with fresh 4T1 breast cancer cells *in vitro* in the presence of prolactin (100 ng/mL) for 48 h. Knockdown of the LF*Prlr* in these sorted, naturally activated Tregs was complete (from a mean relative expression derived from RNAseq of 0.732 in 6 animal tumors from Control SMO-treated animals to 0 in LFPRLR SMO-treated animals). After the 48 h co-culture, the non-adherent tumor Tregs were removed and the expression of mesenchymal genes in the adherent 4T1 cells was analyzed by qRT-PCR (*i.e.* in this experiment, the analyzed 4T1 cells were not themselves treated with LFPRLR SMO or Control SMO). In 4T1 cells co-cultured with tumor Tregs collected from LFPRLR SMO-treated mice, the expression of all 4 examined mesenchymal markers was lower than in 4T1 cells co-cultured with tumor Tregs collected from Control SMO-treated mice (Fig. 4A). Thus, either a direct impact of LFPRLR SMO treatment on tumor Tregs or an indirect effect on the Tregs due to LFPRLR SMO treatment on other tumor cell types or elsewhere *in vivo* negatively impacted the ability of tumor Tregs to drive EMT in epithelial tumor cells.

To further dissect this result, we asked whether the prolactin in the incubation, which was included to replicate the *in vivo* situation, had any direct effect on mesenchymal gene expression in the 4T1 cells or whether the whole of the difference between control and LFPRLR SMO-treatment was *via* the Tregs. 4T1 cells were therefore treated with prolactin in the absence of Tregs and the expression of mesenchymal markers was analyzed. In the absence of Tregs, treatment of 4T1 cells with prolactin significantly stimulated the expression of *Fn1* (*Fibronectin)* and *Snai2 (Snail2),* reduced expression of *Vim (Vimentin)* and had no effect on *Twist1* (Fig. 4B). The *Fn1* and *Snai2* results indicate that some of the *in vitro* co-culture response was due to a direct effect of prolactin on the 4T1 cells. However, the degree of response supports an additional indirect effect *via* the Tregs: compare 2-fold difference with prolactin in Fig. 4B with 10-fold difference in Fig. 4A for *Fn1*, and 4- fold difference with prolactin in Fig. 4B with 12-fold difference in Fig. 4A for *Snai2*. For *Vim*, prolactin has the opposite effect in the presence *versus* absence of Tregs (compare panels in 4A with 4B). For *Twist1*, prolactin had no direct effect on the 4T1 cells. Therefore, all effects of LFPRLR SMO for both *Vim* and *Twist1* in Fig. 4A were *via* Tregs.

As the tumor Tregs in Fig. 4A were collected from animals treated systemically with the LFPRLR SMO or Control SMO, the change of function in tumor Tregs with LFPRLR SMO treatment could possibly have been secondary to changes in other cells within the tumor microenvironment. To investigate whether the loss of ability to promote a mesenchymal phenotype indeed came from knockdown of the LFPRLR in Tregs themselves, we isolated tumor Tregs from untreated Foxp3^+^EGFP Balb/c mice and *ex vivo* treated with 1 μM Control SMO or LFPRLR SMO for 48 h. These tumor Tregs therefore came from the same tumor microenvironment *in vivo* and only differed in LFPRLR expression *ex vivo*. These *ex vivo* treated tumor Tregs were then co-cultured with 4T1 cells as before and the expression of mesenchymal genes in the 4T1 cells was then examined. The expression of 3 of the mesenchymal genes in 4T1 cells was lower when cultured with LFPRLR SMO-treated tumor Tregs *versus* Control SMO-treated tumor Tregs (Fig. 4C), as was the case with *in vivo* treatment (Fig. 4A). The fourth gene, *Snai2* showed a similar trend, but the difference was not statistically significant (Fig. 4C). Thus, a significant portion of the stimulatory effect of Tregs on the expression of mesenchymal genes in 4T1 cells is *via* an effect of prolactin directly on the Tregs.

### LFPRLR SMO treatment does not affect a classical measure of Treg immune-suppression

**Fig. 5:**
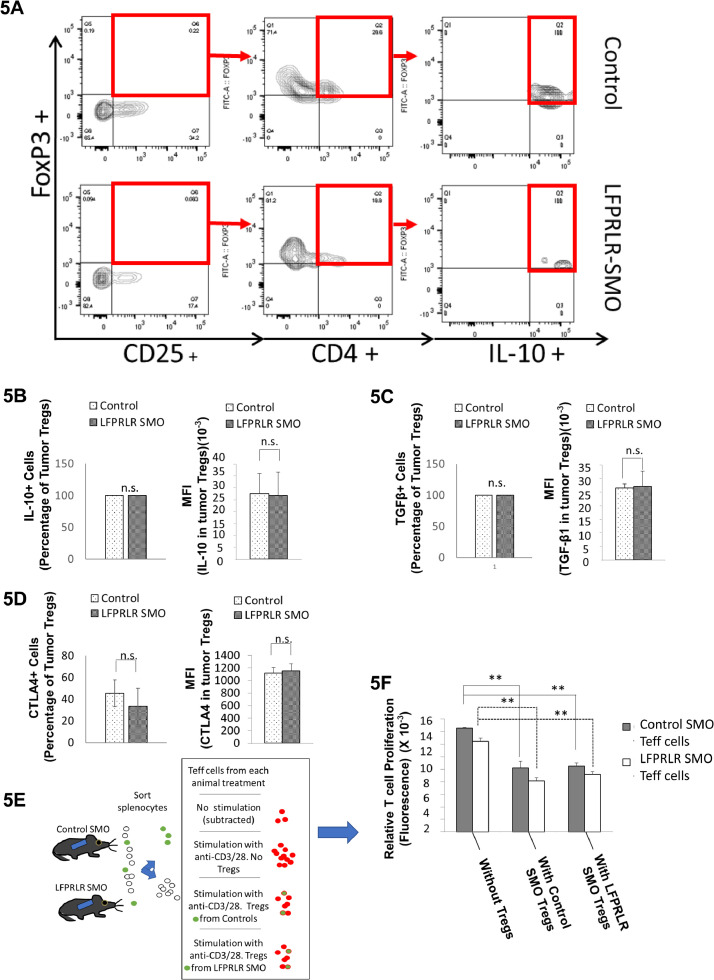
Effects of LFPRLR SMO Treatment on Tumor and Splenocyte Tregs. An example of flow cytometry gating showing IL-10 in tumor Tregs from Control SMO- or LFPRLR SMO-treated mice 12 days after inoculation of ten million 4T1 breast cancer cells (A). Although the number of Tregs was decreased, neither the percentage of tumor Tregs that produce IL-10 (B) or TGF-β1 (C) nor the production of IL-10 (B) or TGF-β1 (C) per cell, assessed as mean fluorescence intensity (MFI), was affected by LFPRLR SMO treatment. In addition, neither the percentage of cells with surface CTLA-4, nor the amount of surface CTLA-4 per tumor Treg was affected (D). Diagram of an experiment to determine the long-term effect of LFPRLR knockdown on the proliferation of splenic T effector (Teff) cells and the regulatory ability of Tregs. After sorting out Tregs, Teffs were stimulated with anti-CD3/CD28 in the absence and presence of Tregs from control SMO- or LFPRLR SMO-treated Foxp3^+^EGFP mice (E). LFPRLR SMO had no effect on the proliferation of anti-CD3/CD28 activated-splenocytes in the absence of Tregs (F, left 2 bars). Co-culture of LFPRLR SMO-treated splenic Tregs with anti-CD3/CD28-activated splenocytes had an indistinguishable suppressive effect compared to Control SMO-treated splenic Tregs. (For A-D, *n* = 12 for LFPRLR SMO treatment and *n* = 24 for Control SMO treatment). Panel F was derived from 6 replicates at 1, 2,3 and 5 months of treatment. Data were indistinguishable and were therefore combined. Data are presented as mean ± SD. (** *p*<0.01).

Tumor Tregs contribute to immune tolerance of tumor parenchyma. We therefore asked whether LFPRLR SMO had any impact on the immune suppressive function of tumor Tregs. Treatment with the LFPRLR SMO showed no effect on tumor Treg IL-10 or TGF-β1 content or cell surface CTLA-4 after 12 days of treatment, either as a proportion of CD4^+^ CD25^+^ Foxp3^+^ cells that expressed these molecules or the MFI for each (Fig. 5B-D). At the mRNA level, RNAseq analysis (GEO: GSE111329) of sorted Tregs again showed no effect of LFPRLR SMO on expression of the immunosuppressive cytokines, *Tgf-β1* and *Il10.* However, *Ctla-4* relative mean expression from 6 animals was reduced from 2.235 in Control SMO-treated to 0.156 in LFPRLR SMO-treated. At the same time, there was no significant effect on whole tumor *Ctla-4* expression.

To determine whether LFPRLR SMO affected the ability of peripheral Tregs (*i.e.* those not within the tumor) to suppress effector cell proliferation, we treated non-tumor bearing animals (Foxp3^+^EGFP Balb/c mice) for up to 5 months with Control SMO or LFPRLR SMO and then isolated splenocytes. This experiment is diagramed as Fig. 5E. After sorting out the Tregs *via* EGFP positivity, we asked 1) whether there was any effect of LFPRLR SMO treatment on the ability of effector cells to proliferate in response to anti-CD3/CD28 and 2) whether treatment with LFPRLR SMO altered the ability of splenic Tregs to inhibit this response. As seen in Fig. 5F, there was no difference in proliferation of splenocytes, from which the endogenous Tregs had already been removed, in response to anti-CD3/CD28 stimulation whether the splenocytes came from animals treated with Control SMO or LFPRLR SMO (left pair of bars).

To test the regulatory capacity of the Tregs from Control SMO- or LFPRLR SMO-treated animals, sorted Tregs from spleens of treated animals were incubated with splenocytes (minus Tregs) from either Control SMO- or LFPRLR SMO-treated animals. The proliferation of splenic cells collected from Control SMO-treated mice (gray bar) was inhibited when co-cultured with Tregs either from Control SMO- or LFPRLR SMO-treated mice. The same degree of inhibition was observed for proliferation of splenocytes from LFPRLR SMO-treated mice (white bar) when co-cultured with Tregs either from Control SMO- or LFPRLR SMO-treated mice. The results were indistinguishable at 1, 2, 3 and 5 months of treatment. Therefore, LFPRLR SMO had no effect on peripheral effector cell proliferation or on the ability of Tregs to inhibit proliferation in the Foxp3^+^EGFP Balb/c mice.

**Fig. 6:**
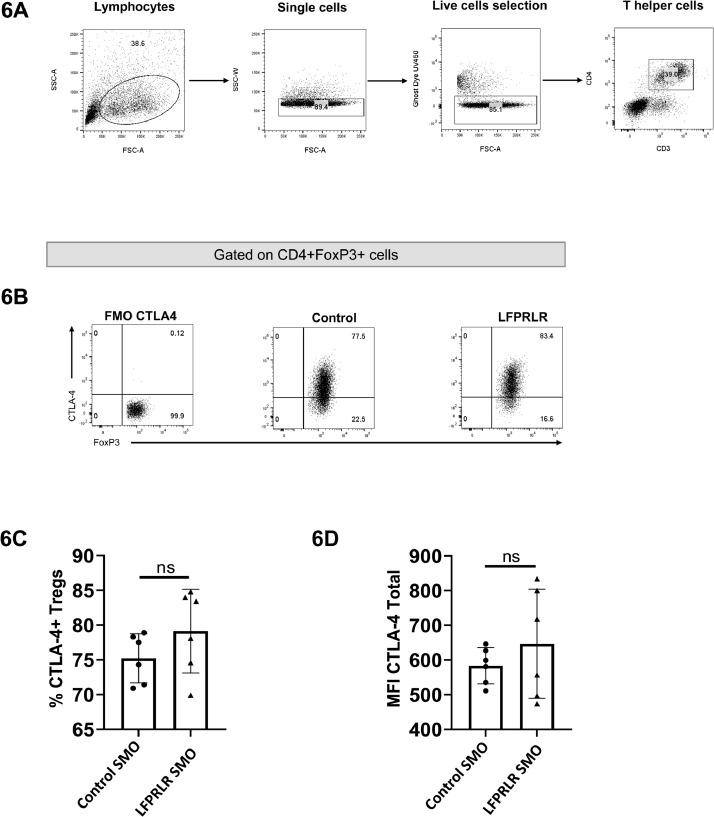
Effect of LFPRLR SMO on Splenic Treg CTLA-4. MRL-lpr mice without tumors were treated with Control SMO or LFPRLR SMO for 56 days. Splenocytes were isolated and analyzed by flow cytometry. Panels A and B show the gating strategy, panel C, the percentage of CTLA-4^+^Tregs, and panel D, the MFI for total (intracellular and surface) CTLA-4 per Treg cell. *n* = 6 animals for each group.

To examine potential extra-tumor effects further and because of the intra-tumor effect on Treg *Ctla-4* expression, we used MRL-lpr mice. These mice have about 5-fold normal prolactin levels at 9 weeks of age [36] and their Tregs exhibit full regulatory activity *in vitro* [37]. If LFPRLR signaling directly regulates CTLA-4 expression, then cells from these animals are more likely to exhibit greater recycling and turnover of CTLA-4. Flow cytometric analysis of splenic Tregs after 2 months of treatment with Control SMO or LFPRLR SMO showed no difference in expression of total (surface and intracellular) CTLA-4 (Fig. 6).

### Effect of LFPRLR SMO on cytokine/chemokine expression in tumor Tregs

**Fig. 7:**
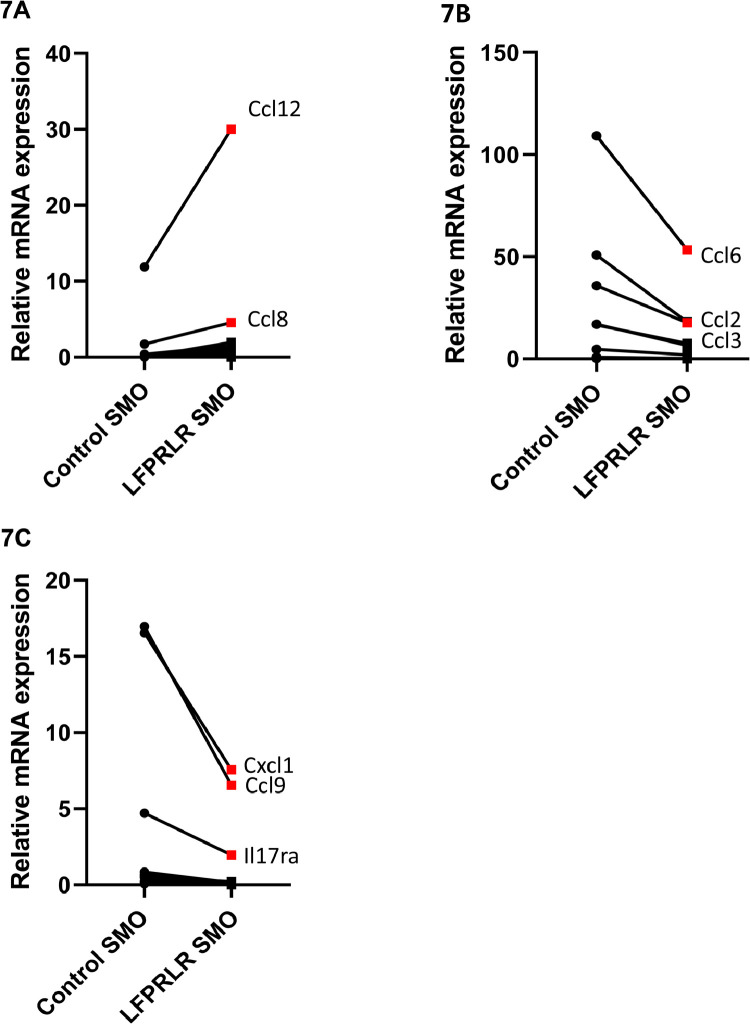
RNAseq Analysis of Tumor Treg Cytokine/Chemokine Expression. RNAseq expression analysis of Tregs isolated from tumors from Control SMO- and LFPRLR SMO-treated mice showed increases (A) and decreases (B, C) in cytokine/chemokine/receptor expression. Different scales are used to more clearly illustrate the degree of change. Those in panel A not labeled and below *Ccl8* in relative expression are in decreasing expression order *Ccl5, Cxcl10, Il18bp, Ccl11, Cxcl13, Il21r, Il1rap, Il1r1, Tgfα, Ccl22, Cxcl9, Il24, Il5rα, Tgfβ2*, and *Il22rα1*. Those decreased by LFPRLR SMO and below *Il17rα* in expression are in order of decreasing expression *Il2rα, Il15, Cxcl11, Il15rα, Il23α, Il27rα, Il12rβ1, Ccr8, Il3rα, Il34, Cxcl15, Ccr4, Cxcr3, Il17rc* and *Ebi3*. Gene names are not italicized in the figure in order to increase readability. Data are the mean of 6 animals treated with Control SMO and 6 treated with LFPRLR SMO.

Having established that LFPRLR SMO treatment did not affect TGF-β1 or IL-10 production by tumor Tregs, we used RNAseq analysis of tumor Tregs to gain some unbiased insight into which other secreted factors could possibly be responsible for the reduced ability of Tregs to promote epithelial to mesenchymal transition after LFPRLR SMO treatment. Expression of 17 cytokine/chemokine/receptor genes increased by a factor of 2 or more, and 24 were decreased (see figure legend for complete list). Those most highly expressed are shown in red (Fig. 7). Quantitatively the most important were downregulation of *Ccl6, Ccl2* and *Ccl3* and upregulation of *Ccl12*.

**Fig. 8:**
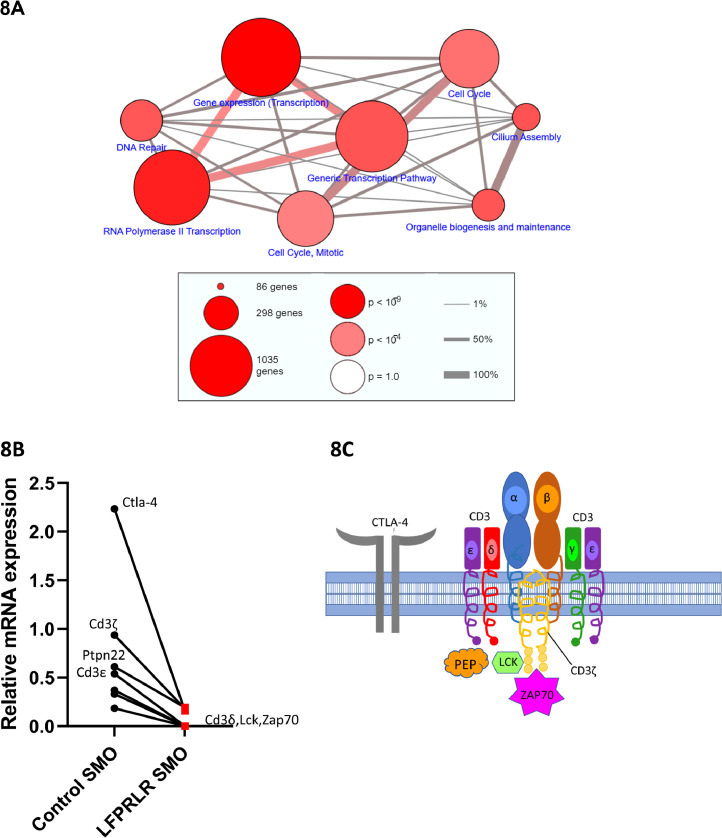
Other Transcripts Downregulated in Tumor Tregs. Pathway analysis showing the top 8 most statistically significant (A). The size of the circles represents the relative number of genes and the color intensity of the circles, the relative significance. The thickness of the interconnecting lines indicates the degree of overlap in the terms associated with the circles. Further pathway analysis identified a pathway described as “Translocation of ZAP70 to the immunological synapse” with a *p* value of 4.5 × 10^−^4. Downregulation of genes within this category is shown (B) and their arrangement in the membrane and cytoplasm is cartooned (C). The CD3 complex is composed of δ, ε, γ, and ζ proteins and only γ was not affected by LFPRLR SMO treatment. Lck is a Src-like protein tyrosine kinase that, in response to engagement of the TCR-CD3 complex, phosphorylates regions of the CD3 intracellular domains. This, in turn leads to recruitment and activation of the CD3 ζ chain-associated protein kinase, ZAP70. PEP (PEST domain-enriched tyrosine phosphatase) is the protein product of the mouse *Ptpn22* gene. It regulates the activity of Src like kinases. CTLA-4 normally downregulates CD80 and CD86 expression on antigen-presenting cells, thereby limiting the ability of antigen-presenting cells to stimulate Teff cells.

To gain insight into other effects of LFPRLR SMO treatment on tumor Tregs, pathway analysis (ConsensusPathDB) of transcripts downregulated in Tregs by LFPRLRSMO treatment was performed (Fig. 8A). Shown are the 8 most statistically significant pathways. There were major inhibitory effects on gene transcription, cell cycle and DNA repair genes, and on genes traditionally recognized as being involved in the formation of cilia, but now also recognized as being involved in the formation of an immunological synapse [38]. Also, although composed of fewer transcripts, another pathway of significance to specific function and downregulated by LFPRLR SMO (*p* = 4.5 × 10^−^4) was entitled “translocation of ZAP70 to the immunological synapse”. Those genes were *Ctla-4*, as previously mentioned, *Cd3ζ, Ptpn22, Cd3ε, Cd3δ, Lck* and *Zap70* (Fig. 8B); their association at the T cell receptor complex is diagrammed in Fig. 8C.

## Discussion

We have determined that knockdown of the LFPRLR increased survival in an immunocompetent, but not immunodeficient, breast cancer model. While there could be several reasons for this difference, here we have focused on an aspect of adaptive immunity, *viz.* how LFPRLR knockdown affected tumor-Treg interactions. Tumor cells are protected from effector T cell action by the presence of Tregs. Given that Tregs are overrepresented in tumors [3], [4], [5], [6], reducing their number and/or activity within tumors is an important goal in the treatment of cancer. This goal was accomplished by treatment with the LFPRLR SMO. Knockdown of the LFPRLR not only reduced Treg percentages in the primary tumor, but also in the draining lymphnode, and maybe also in the liver, which is a major site of metastasis. Through a variety of analyses, we were able to conclude that LFPRLR knockdown decreased production of the Treg chemoattractant, CCL17, by tumor parenchyma. At the same time, there was no effect on 4T1 production of CCL22. Thus, one would anticipate reduced, but not eliminated Treg recruitment to 4T1 cells, as we observed in the tumors. Nevertheless, a reduction in the percentage of Tregs sets the stage for enhanced T cell responses to the tumor. Consistent with this scenario, we observed increased CD4^+^ and CD8^+^ effector cell representation in the tumors but unlike previous analyses, where tumors were examined after 42 days of treatment [24], only the CD4^+^ cells were activated at the earlier timepoint of 28 days.

A larger number of Tregs in primary tumors is associated with increased metastasis in a number of different malignancies [7], [8], [9], [10]. Importantly, our co-culture results demonstrate not just an association, but a causal relationship between Tregs and promotion of metastasis, at least in regard to EMT. *In vivo*, prolactin has the capacity to affect function of the tumor parenchyma both directly and indirectly *via* the Tregs. A combination of *ex vivo* and *in vitro* experiments demonstrated an effect of prolactin, as well as substances derived from the Tregs in response to prolactin, on the EMT process. However, the larger influence of prolactin (working through the LFPRLR) in all cases was *via* an effect on Tregs. RNAseq analysis of tumor Tregs revealed 24 cytokines/chemokines/receptors decreased in expression by LFPRLR knockdown. Among these, CCL6, CCL2, and CCL9 have each been shown to promote EMT [39], [40], [41]. Based on the current literature, therefore, these chemokines seem likely candidates, but establishment of them, or indeed one or more of the other 21 cytokines downregulated by LFPRLR SMO, as important to the promotion of EMT in intact tumors will require substantial further investigation. For now, what has been unequivocally established is that prolactin has direct effects on tumor Tregs that cause them to promote EMT.

A point of potential controversy in our results stems from a publication that described prolactin as an agent that reduces rather than enhances EMT, although this was defined by only one EMT marker, vimentin [42]. A careful examination of our results does not disagree with this assessment if one is examining cancer cells in isolation and focuses on vimentin as the only mesenchymal marker. However, in the presence of Tregs, prolactin has the opposite effect on vimentin expression and additionally increases expression of three other mesenchymal genes.

While reducing the number of Tregs within the primary tumor and reducing the ability of the remaining Tregs to promote EMT, LFPRLR SMO treatment did not affect the ability of Tregs to produce the classical immune suppressive cytokines, IL-10 and TGF-β1. At the 12-day time point, there was also no effect on cell surface CTLA-4. However, unlike IL-10 and TGF-β1 mRNA, *Ctla-4* transcripts were largely eliminated at 12 days such that at later timepoints, one would expect a diminution of cell surface CTLA-4 and subsequent diminution of Treg inhibition of effector cell clearance of tumor cells. In addition, RNAseq analysis in the sorted tumor Tregs suggests inhibition of the formation of immune synapses both by lowered expression of components of the T cell receptor complex, signaling molecules, and structural entities that create polarized secretion.

Although all data herein support inhibited function of the tumor Tregs, there was no evidence of inhibited function of splenic Tregs since there was no change in a) CTLA-4 in splenic Tregs after 2 months of treatment in a model more likely to show changes, or b) in the ability of splenic Tregs to inhibit proliferation of T effector cells after 1–5 months of treatment. The effect of LFPRLR SMO on tumor Treg suppressive function must therefore be indirect and a consequence of an altered tumor microenvironment. This finding underscores the importance of examining drug effects within the complexities of a tumor in orthotopic and immunocompetent model systems.

Based on the normal activities of effector cells and Tregs in the spleen, one would predict that treatment with the LFPRLR SMO would have reduced adverse inflammatory and autoimmune effects compared to other cancer immunotherapeutics. In this regard, our results are not at odds with previous conclusions about the role of elevated prolactin in autoimmune disease since prolactin inhibits the immune-suppressive role of Tregs on T effector cell proliferation [32]. In the absence of the LFPRLR, Treg function would not be inhibited.

In conclusion, LFPRLR SMO improves survival in an immunocompetent model, increases the capacity for anti-tumor immunity through increased numbers of effector cells and reduced numbers of Tregs in the tumors, and reduces both the metastasis-promoting ability of the Tregs still present and likely also their suppressive function. Furthermore, this is accomplished without apparent negative effects on the ability of splenic Tregs to suppress proliferation of effector T cells.

## Declaration of Competing Interest

The authors declare no potential conflicts of interest

## Appendix Supplementary materials

Image, application 1
