# Supplementary material for: Prolactin enhances T regulatory cell promotion of breast cancer through the long form prolactin receptor
Source: Transl Oncol. 2021 Aug 7;14(11):101195. doi: 10.1016/j.tranon.2021.101195 (PMC8358703; doi:10.1016/j.tranon.2021.101195)
Supplement: Supplementary file 1 [file mmc1.docx]

**Supplementary Material**

**
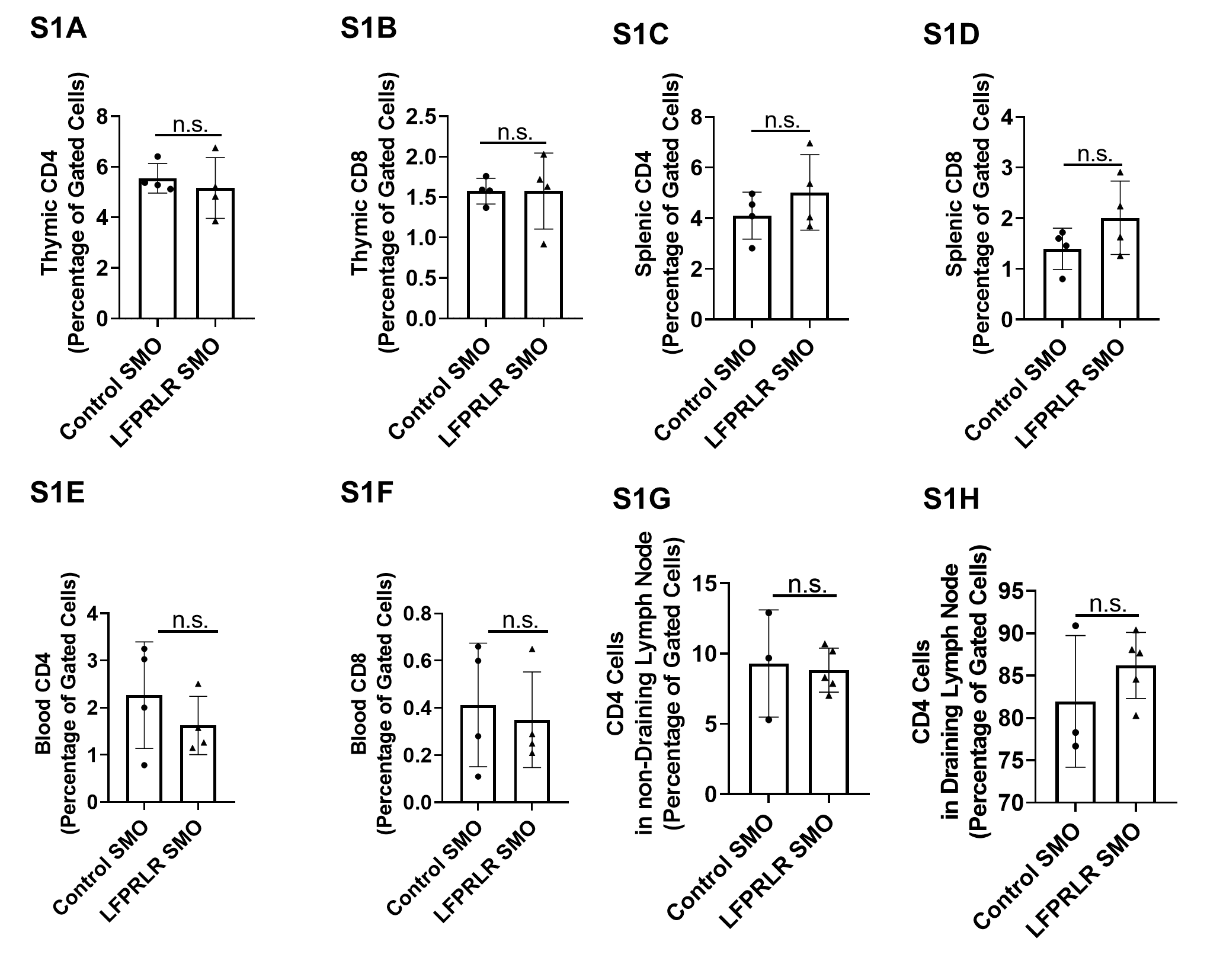
**

**Supplementary Figure 1: Systemic Effector T cells Were Not Affected by LFPRLR SMO Treatment.**

Tumors were produced in 8-week-old female Foxp3^+^EGFP Balb/c mice by injecting 10 million 4T1 syngeneic breast cancer cells into the mammary fat pad. Thymus, spleen, blood, draining or non-draining Inguinal lymph nodes were collected at 28 days. There were no observed differences in effector CD4+ or CD8+ T cells in all examined tissues (A-H). Results were derived from 3-5 mice. Data are presented as mean ± SD.


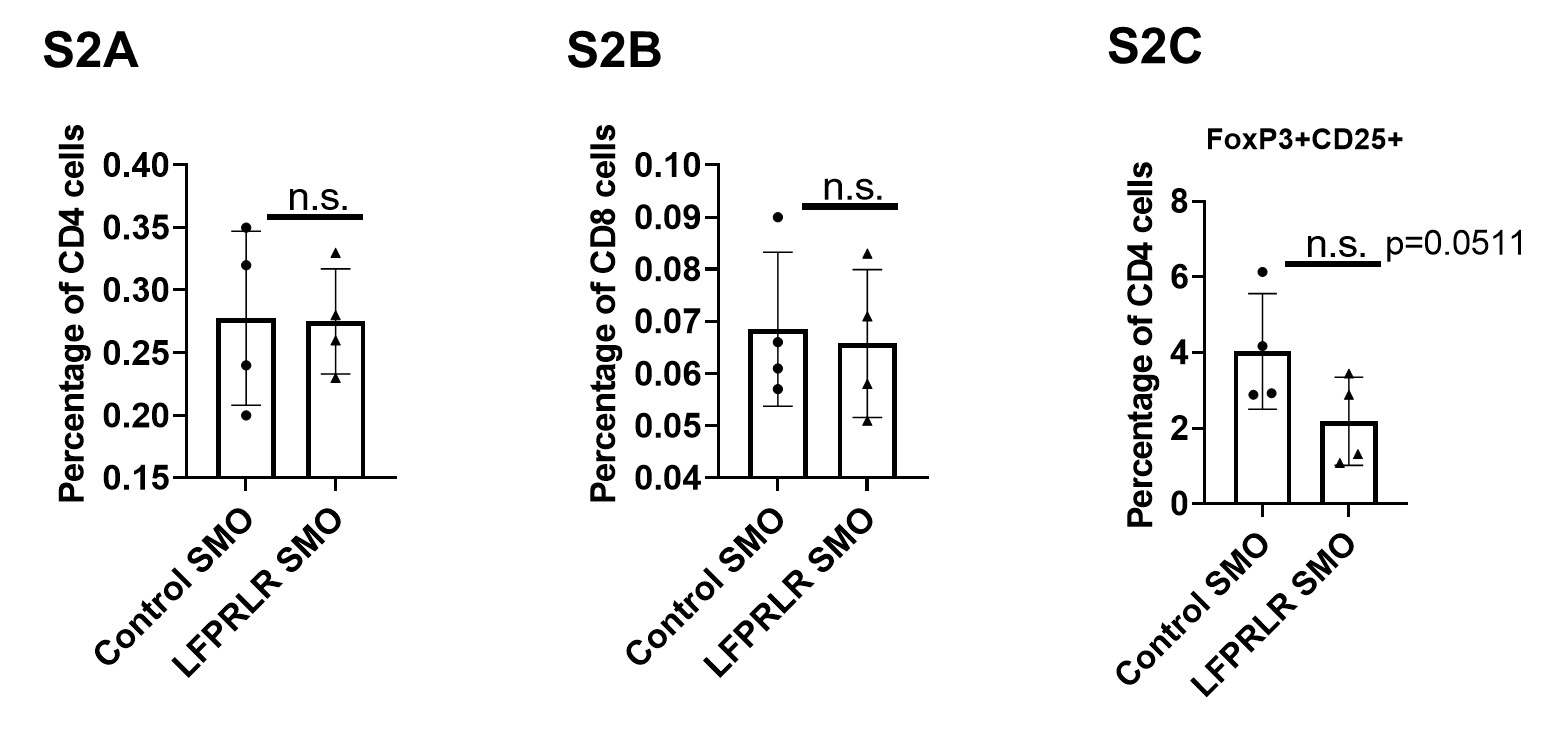


**Supplementary Figure 2: Metastatic Liver Effector and Treg Cells.**

Tumors were produced in 8-week-old female Foxp3^+^EGFP Balb/c mice by injecting 10 million 4T1 syngeneic breast cancer cells into the mammary fatpad. T cells in metastasis-bearing livers were examined at 12 days by flow cytometry. There were no observed differences in effector CD4+ or CD8+ T cells (A and B). However, there was a strong trend towards lower Tregs in liver (C). Results were derived from 4 mice (*n* =4). Data are presented as mean ± SD.

| **Table I Antibodies used in flow cytometric analysis** | | |
| --- | --- | --- |
| Antibody | company | cat# |
| CD4 | TONBO bioscience | 25-0042-U100 |
| CD25 | TONBO bioscience | 60-0251-U100 |
| CD8 | TONBO bioscience | 55-0081-U100 |
| IL-2 | TONBO bioscience | 20-7021-U100 |
| CTLA-4 | TONBO bioscience | 20-1522-SU05 |
| IFNγ | TONBO bioscience | 50-7311-U100 |
| TGF-β | BioLegend | 141410 |
| IL-10 | ebioscience | 45-7101-82 |

| **Table II** **Primer list for qRT-PCR** | |
| --- | --- |
| Gene | primer sequence |
| *Vim* | F : ACTGCAGGAGCTGAATGACC |
|  | R : AAGGTCAAGACGTGCCAGAG |
| *Snai2* | F : CGCCTGGACCGTTATCCG |
|  | R : CTGCCGACGATGTCCATACA |
| *Twist1* | F : CCCACCCCACTTTTTGACGAAG |
|  | R : GCCAGTTTGATCCCAGCGTT |
| *Fn1* | F : TGTGAGCCGGACAACTTCTG |
|  | R : CCTAGGTAGGTCCGTTCCCA |
| *Ccl17* | F : ATCAGGAAGTTGGTGAGCTGG  R : TGGTCACAGGCCGTTTTATGT |
| *Ccl22* | F : ACCCATTCTTGCTTCCCTGG  R : CTGGCACTGTCAATCCCTGT |
| *Gapdh* | F: TGCACCACCAACTGCTTAG  R: GGATGCAGGGATGATGTTC |
|  |  |
|  |  |
